# Process Optimization of the Hot Stamping of AZ31 Magnesium Alloy Sheets Based on Response Surface Methodology

**DOI:** 10.3390/ma16051867

**Published:** 2023-02-24

**Authors:** Pengjing Zhao, Qi Wu, Yo-Lun Yang, Zhanghua Chen

**Affiliations:** 1 Faculty of Materials and Manufacturing, Beijing University of Technology, Beijing 100124, China; 2 Graduate Institute of Manufacturing Technology, National Taipei University of Technology, Taipei 106344, China; 3 School of Mathematics and Physics, University of Science and Technology Beijing, Beijing 100083, China

**Keywords:** magnesium alloy sheet, hot stamping, response surface analysis, numerical simulation

## Abstract

Hot stamping is an important manufacturing process for sheet metal parts. However, it is easy to produce defects such as thinning and cracking in the drawing area during the stamping process. In this paper, the finite element solver ABAQUS/Explicit was used to establish the numerical model of the magnesium alloy hot-stamping process. The stamping speed (2~10 mm/s), the blank-holder force (3~7 kN), and the friction coefficient (0.12~0.18) were selected as the influencing factors. Taking the maximum thinning rate obtained through simulation as the optimization objective, the response surface methodology (RSM) was applied to optimize the influencing factors in sheet hot stamping at a forming temperature of 200 °C. The results showed that the maximum thinning rate of sheet metal was most influenced by the blank-holder force, and the interaction between the stamping speed and the blank-holder force/friction coefficient had a great influence on the maximum thinning rate. The optimal value of the maximum thinning rate of the hot-stamped sheet was 7.37%. Through the experimental verification for the hot-stamping process scheme, the maximum relative error between the simulation and the experimental results was 8.72%. This proves the accuracy of the established finite element model and the response surface model. This research provides a feasible optimization scheme for the analysis of the hot-stamping process of magnesium alloys.

## 1. Introduction

With the increasingly severe energy crisis and emission limits, lightweight manufacturing has become the inevitable trend in the future development of automobiles. Magnesium alloys show good application prospects in the automobile field for their excellent properties such as high strength, low density, excellent heat dissipation, and electromagnetic shielding [1,2]. However, as a result of magnesium alloy’s hexagonal close-packed (hcp) crystal structure, its plastic deformation ability at room temperature is poor, and tensile cracking and wrinkling defects easily occur [3]. Previous research [4] has shown that when a magnesium alloy sheet is heated to above 200 °C, the first-order conical surface of the crystal structure and {1011} and {1021} slip systems are activated, and the plastic deformation capacity greatly improves. Thus, the hot-stamping technology of magnesium alloy sheets has attracted much attention [5].

Improvements in the hot-stamping process are often based on repeated testing, which is an extremely expensive and time-consuming process [6]. It is therefore necessary to reduce manufacturing-process costs by minimizing design time and physical tests. In recent decades, the combination of finite element simulation [7], experimental techniques [8,9], and optimization methods [10,11] has provided a promising alternative to the optimal hot-stamping process design. For instance, Xiao et al. [12] proposed a multi-objective stochastic method to determine the optimal parameters of the hot-stamping process and further studied the effect of these parameters on processing quality in a limited range. Based on the RSM and non-dominated sorting genetic algorithm (NSGA-I), Bao et al. [13] performed a multi-objective optimization procedure for the partition temperature of a hot-stamped steel sheet. Lei et al. [14] developed a novel finite element model coupled with thermoelastic–plastic behavior for the hot stamping of blank patchwork to predict the stamping results and obtain the optimum process parameters. A constitutive equation developed by Namklang et al. [15] was combined with finite element results to assess the local deformability of hot-stamping products. Gao et al. [16] proposed a novel multi-objective optimization method for improving the forming quality and energy consumption in the hot-stamping process. Cui et al. [17] used a three-dimensional forming limit diagram (FLD), which considered phase transformations, to evaluate the forming property of the wrinkling of high-strength steel during hot stamping. A finite element model coupled with a thermal–mechanical phase was established by Quan et al. [18] to examine the effect of hot-stamping process variables on the evolution of the phase field. Hu et al. [19] derived a thermal–mechanical constitutive equation combined with material damage to describe the hot-stamping behavior of high-strength steel and further studied the effects of the blank-holder force and contact relationship on the punch force, crack initiation, and formability.

In the above research, numerous optimization studies mainly focus on the hot-stamping process for aluminum alloy/steel sheets, and there is not enough research on a hot-stamping process for magnesium alloy sheets. In addition, there are many factors affecting formability, mainly including the blank-holder force, strain rate, lubrication effects, and die clearance during the hot-stamping process [20]. Due to the mutual restriction of the process variables, it is crucial to obtain the combination of technical parameters that can guarantee the forming quality and performance of the forming parts. Therefore, the aim of this work is to better understand the effects of process parameters on magnesium alloy sheet thinning and optimize the forming process to minimize the sheet thinning of hot-stamped sheets.

The present paper is organized as follows: In Section 2, the constitutive model suitable for magnesium alloy sheet forming is introduced, and a finite element model of the hot-stamping process of a magnesium alloy sheet is established and verified. In Section 3, taking the maximum thinning rate of the hot-stamped sheet as the optimization objective, the RSM is applied to investigate the impact of three process parameters on the maximum thinning rate of the sheet. In Section 4, the accuracy of the numerical model and the response surface model is verified using hot-stamping experiments. Finally, the conclusions are summarized in Section 5.

## 2. Numerical Analysis of the Hot-Stamping Process

### 2.1. Constitutive Model

Barlat et al. [21] proposed a yield criterion applicable to the plane stress state, which is called the YId2000 yield criterion, to better describe the plastic anisotropy behavior of magnesium, aluminum, and other metal sheets. This criterion represents the anisotropy properties of materials through two linear transformations of the Cauchy stress tensor. The in-plane anisotropy behavior of metal materials can be better described compared with the well-known Hill yield criterion, and its yield surface function is expressed as

(1) $$\phi ={\left|S_{1}^{\prime }-S_{2}^{\prime }\right|}^{a}+{\left|2S_{2}^{\prime \prime }+S_{1}^{\prime \prime }\right|}^{a}+{\left|2S_{1}^{\prime \prime }+S_{2}^{\prime \prime }\right|}^{a}=2{\bar{\sigma }}^{a}$$

where

(2) $$\left\{ \begin{array}{l} S^{\prime }=L^{\prime }\cdot \sigma \\ S^{\prime \prime }=L^{\prime \prime }\cdot \sigma \end{array}\right.$$

where $S^{\prime }$ and $S^{\prime \prime }$ are the linear transformations of stress tensors, and $S_{1}^{\prime }$, $S_{2}^{\prime }$, $S_{1}^{\prime \prime }$, and $S_{2}^{\prime \prime }$ are the principal values of the stress tensor.

(3) $$L^{\prime }=\left(\begin{array}{l} L_{11}^{\prime }\\ L_{12}^{\prime }\\ L_{21}^{\prime }\\ L_{22}^{\prime }\\ L_{66}^{\prime } \end{array}\right)=\left(\begin{array}{ccc} 2/3 & 0 & 0\\ -1/3 & 0 & 0\\ 0 & -1/3 & 0\\ 0 & 2/3 & 0\\ 0 & 0 & 1 \end{array}\right)\left(\begin{array}{c} \alpha_{1}\\ \alpha_{2}\\ \alpha_{7} \end{array}\right)$$

(4) $$L^{\prime \prime }=\left(\begin{array}{c} L_{11}^{\prime \prime }\\ L_{12}^{\prime \prime }\\ L_{21}^{\prime \prime }\\ L_{22}^{\prime \prime }\\ L_{66}^{\prime \prime } \end{array}\right)=\frac{1}{9}\left(\begin{array}{ccccc} -2 & 2 & 8 & -2 & 0\\ 1 & -4 & -4 & 4 & 0\\ 4 & -4 & -4 & 1 & 0\\ -2 & 8 & 2 & -2 & 0\\ 0 & 0 & 0 & 0 & 9 \end{array}\right)\left(\begin{array}{c} \alpha_{3}\\ \alpha_{4}\\ \alpha_{5}\\ \alpha_{6}\\ \alpha_{8} \end{array}\right)$$

where *α_k_* parameters are the independent coefficients of material (for *k* from 1 to 8).

The ductile Lemaitre damage model [22], which is based on a thermodynamic framework, is adopted to accurately describe the damaging behavior of the material. Damage can be accurately quantified using the internal variable *D*, which lies in the range of 0 ≤ *D* < 1 when represented in its scalar form. This variable (*D*) represents the ratio of the damaged area of a unit surface (*S_D_*) to the total surface (*S*): *D* = *S_D_*/*S*.

Based on the hypothesis of strain equivalence, the equivalent stress tensor with damage can be defined as

(5) $$\tilde{\sigma }=\sigma /\left(1-D\right)$$

where ***σ*** is the stress tensor of the material without considering the damage behavior.

The damage strain energy release rate *ψ* is associated with the damage variable *D*, and it can be expressed as follows:

(6) $$\psi =\frac{q^{2}}{2E{\left(1-D\right)}^{2}}\left[\frac{2}{3}\left(1+v\right)+3\left(1-2v\right){\left(\frac{\sigma_{H}}{q}\right)}^{2}\right]$$

where *E* is the Young modulus, *ν* is the Poisson ratio, *q* is the equivalent stress, *σ_H_* is the hydrostatic stress, and *σ_H_*/*q* represents the stress triaxiality of the material.

Accordingly, damage evolution can be expressed as

(7) $$\dot{D}=-\dot{\lambda }\frac{\partial F_{Y}}{\partial Y}=\left(\frac{Y}{S_{0}}\right){\dot{\bar{\varepsilon }}}^{p}$$

where $\dot{\lambda }$ is the plastic multiplier, *F* is the plasticity dissipation potential function, and ${\dot{\bar{\varepsilon }}}^{p}$ represents the equivalent plastic strain rate.

As soon as equivalent plastic strain exceeds a strain threshold *ε_D_*, damage increases by the following formula:

(8) $$\dot{D}=\left\{ \begin{array}{l} 0\\ {\left(-\frac{Y}{S_{0}}\right)}^{b}{\dot{\bar{\varepsilon }}}^{p} \end{array}\right.\begin{array}{l} if\;{\bar{\varepsilon }}_{p}<\varepsilon_{D}\\ f\;{\bar{\varepsilon }}_{p}\geq \varepsilon_{D} \end{array}$$

The macroscopic fracture is then accounted for by a critical damage value *D_c_*. Once the value of *D* reaches *D_c_*, the damage variable *D* is assigned a value of 1, which signifies the failure of the material.

Since the YId2000 yield criterion can better describe the plastic anisotropy behavior of a light alloy sheet than the traditional yield criterion [23,24], the plastic deformation and damage behavior of the AZ31 magnesium alloy sheet was analyzed with the Lemaitre damage model modified using the YId2000 yield criterion. The constitutive model parameters applicable to describing the deformation of the AZ31 magnesium alloy sheet are discussed in the authors’ previous research [25]. The anisotropic yield function of Yld2000 was embedded into the material properties with the help of user subroutine VUMAT, and the numerical calculation for the hot-stamping forming process of the magnesium alloy sheet was realized based on the ABAQUS platform.

### 2.2. Establishment of Finite Element Model

The experimental material was a commercial rolled AZ31 magnesium alloy sheet with a thickness of 0.6 mm, and its chemical composition is listed in Table 1. A rectangular plate with a size of 110 mm × 60 mm was cut off using a wire-cutting machine for the hot-stamping test. The mechanical properties of the AZ31 magnesium alloy sheet are given in the authors’ previous study [26].

**Figure 1:**
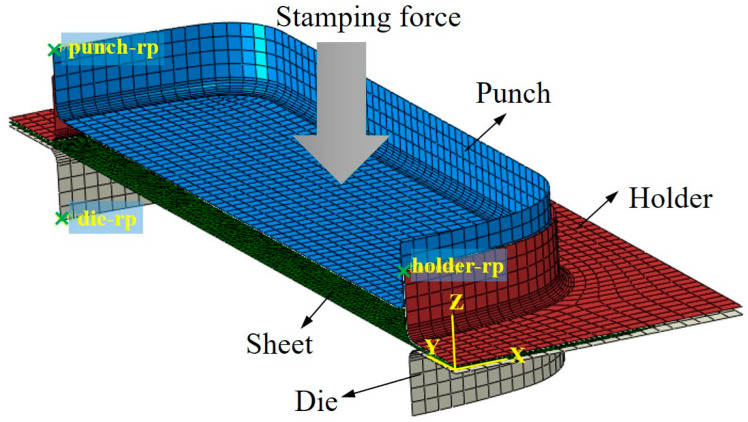
Three-dimensional coupled thermomechanical finite element model of the hot-stamping process.

The hot-stamping process of the AZ31 magnesium alloy sheet was simulated based on the nonlinear finite element solver ABAQUS/Explicit. The three-dimensional coupled thermomechanical finite element model of hot stamping is shown in Figure 1. The whole simulation process of hot-stamping formation was divided into two steps: The holder moved down and made contact with the sheet (step 1), and the punch moved down a fixed distance for hot stamping (step 2). Because changes in temperature and displacement are involved in the forming process, the type of “dynamic, explicit, temperature–displacement” was selected in both analysis steps. The blank magnesium alloy sheet was set as a deformable body, while the die, holder, and punch were set as the rigid body. The Coulomb friction model was adopted for describing the friction between the sheet and dies, and the friction coefficient was 0.15. The sheet was meshed using a four-node thermally coupled doubly curved thin-shell element with reduced integration and hourglass control (S4RT), and five integral points were set in the thickness direction.

Table 2 lists the processing parameters of the AZ31 magnesium alloy sheet during the hot-stamping experiment. Finite element simulation parameters during hot stamping were set in accordance with the actual experimental parameters as follows: The temperature of the environment and the blank were set to 20 °C and 200 °C, respectively; the heat transfer coefficient between the blank and dies was 2000 W/(m^2^ °C); the inelastic heat fraction was 0.9; and the film coefficient was 0.025 W/(m^2^ °C). In addition, the clearance distance between the punch and die was 0.8 mm.

### 2.3. Validation of Finite Element Model

**Figure 2:**
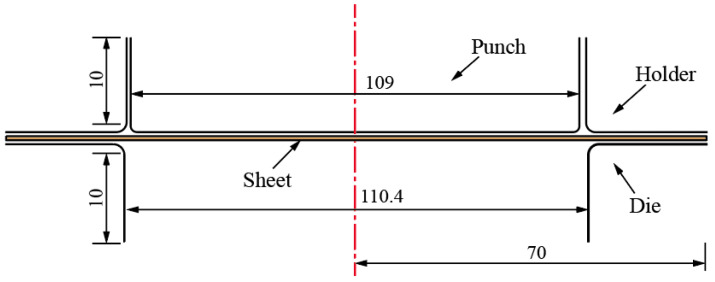
Overall dimensions of hot-stamping dies.

The hot-stamping test of the magnesium alloy sheet was carried out to verify the validity and rationality of the established finite element model of hot stamping. The overall dimensions of the hot-stamping dies are shown in Figure 2. The heated magnesium alloy sheet was placed in the dies, and the hot-stamping experiment was performed after the sheet was located.

**Figure 3:**
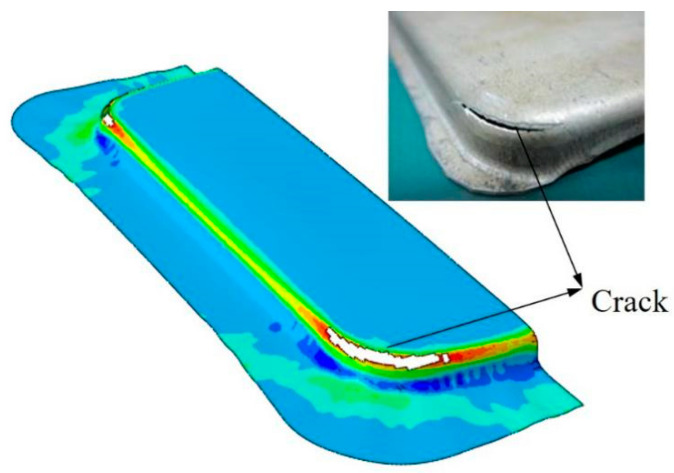
Comparison between results from numerical simulations and hot-stamping experiments.

Comparing the experimental results with the simulation results, it can be observed that a crack appeared in the sheet metal near the corner region of the die, as shown in Figure 3. The fracture location obtained through simulation matched that of the test piece quite well, which indicates the accuracy of the numerical model.

**Figure 4:**
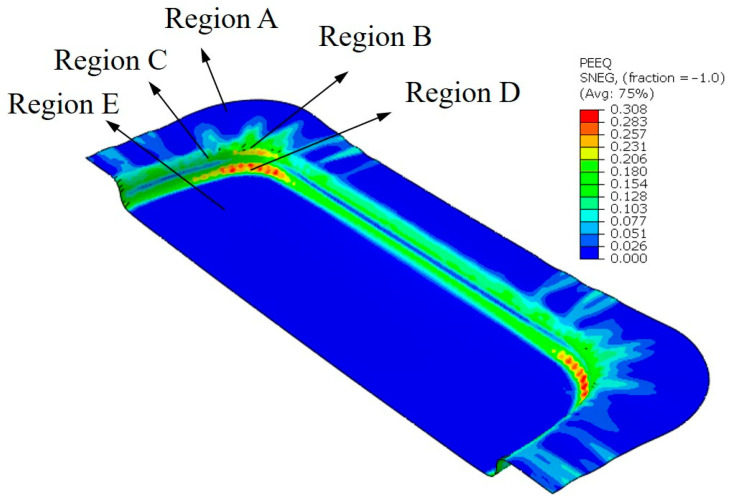
Equivalent plastic strain distribution of magnesium alloy sheet during hot stamping.

The equivalent plastic strain distribution of the hot-stamped sheet obtained through the finite element method is shown in Figure 4. After hot-stamping formation, the fillet region (B) near the punch had a low degree of work hardening, and the material was subjected to greater tensile and compressive stresses in the thickness direction than the material in region A, which was a dangerous area prone to breakage. The straight wall region (C), as the main force transfer region, was affected by radial tensile stress and compressive strain in the thickness direction. In addition, the material in flange region (A) and bottom region (E) was difficult to be replenished in time during hot stamping due to the blank-holder force and the friction of the punch. The sheet near the corner region (D) of the die was prone to thinning due to its large strain. Therefore, the maximum thinning rate of the sheet in region D was selected for subsequent analysis.

## 3. Forming-Parameter Optimization Based on RSM

### 3.1. Establishment of Response Surface Model

The response surface methodology (RSM) is an effective method to solve multi-variable and objective optimization problems. Test groups are designed using reasonable experimental design methods, and certain reliable data can be obtained through experiments or simulations. For fitting the functional relationship between various factors and response values, multiple quadratic regression equations are used. By analyzing regression equations, the best parameter combination is determined. This method is widely used in the metal-forming field because of its characteristics of small computation, the interaction between the analyzed factors, and high convergence.

The hot stamping of magnesium alloy sheets is a nonlinear and large deformation process. An excessive number of optimization parameters and constraints can not only make it difficult to test a large number of samples but can also negatively influence the accuracy of the response surface model and disrupt the optimization process. Therefore, three key forming parameters (stamping speed, blank-holder force, and friction coefficient) were selected as the response variables, and the maximum thinning rate (Y) at the corner of the hot-stamped sheet was taken as the response value. In addition, considering the formability of the magnesium alloy sheet, a punching depth of 6 mm was set for simulation calculation and subsequent experimental verification. The factors and levels for the hot-stamping simulation are listed in Table 3.

Experimental schemes were designed using the Box–Benhnken design (BBD) method in the Design-Expert 12 software after determining the response variables and levels. The hot-stamping process of the magnesium alloy sheet was simulated with the aid of the finite element solver ABAQUS/Explicit. Table 4 shows the finally obtained maximum thinning rate of the stamping sheet under the combined parameters’ scheme.

### 3.2. Variance Analysis of Response Surface Regression Model

The simulation results in Table 4 were fitted using multiple quadratic regression. The final regression model equation of the maximum thinning rate in terms of the actual response factors of the magnesium alloy sheet can be obtained as follows:

(9) $$\begin{array}{ll} Y & =155.5-0.652X_{1}-17.432X_{2}-1380.167X_{3}+0.028X_{1}X_{2}-2.792X_{1}X_{3}\\ & -8.833X_{2}X_{3}+0.065X_{1}^{2}+1.685X_{2}^{2}+5095.833X_{3}^{2} \end{array}$$

where X_1_ is the stamping speed; X_2_ is the blank-holder force; X_3_ is the friction coefficient; and Y represents the maximum thinning rate at the corner of the hot-stamped sheet.

To further verify whether the established response surface model can accurately express the statistical rule between the variables and the optimization objective, variance analysis was carried out for the above equation, as summarized in Table 5. The confidence interval *p*-value of the model < 0.0001 for the equation of the maximum thinning rate of the magnesium alloy sheet was considered extremely significant, indicating that this model could be used in this experiment. The lack of fit > 0.05 was not significant, indicating that the model was reasonable in the regression region and could be used to predict the maximum thinning rate of the magnesium alloy sheet. The multivariate correlation coefficient R^2^ was 0.9912, and the correction coefficient R^2^(Adj) was 0.9799, which indicated that the model fitted well, and the prediction was close to the actual value.

It can also be concluded from Table 5 that the influence order of the three response factors on the maximum thinning rate was as follows: blank-holder force (X_2_) > friction coefficient (X_3_) > stamping speed (X_1_). In addition, the model also characterized the interaction relationship between the three response factors. The interaction terms X_1×2_ and X_1×3_ had a significant impact on the maximum thinning rate, indicating that the stamping speed and the blank-holder force/friction coefficient had an obvious interaction. The interaction term X_2×3_ had no significant effect on the maximum thinning rate, indicating that there was no significant interaction between the two groups of factors.

**Figure 5:**
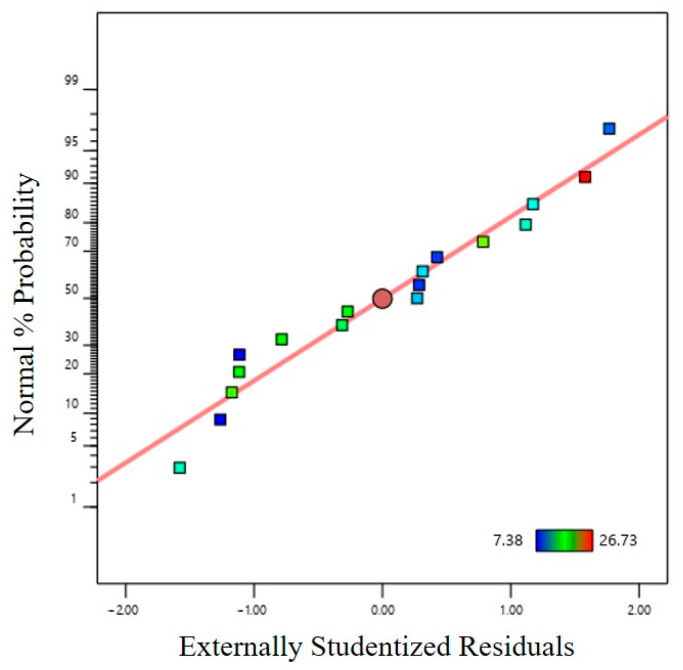
Normal probability distribution of residual.

Figure 5 shows the normal distribution probability of the residual for the maximum thinning rate. The normal distribution probability distribution of the residual is close to a straight line which is fitted well, indicating that the equation between the independent variable and the response variable is highly reliable as a result of regression analysis.

**Figure 6:**
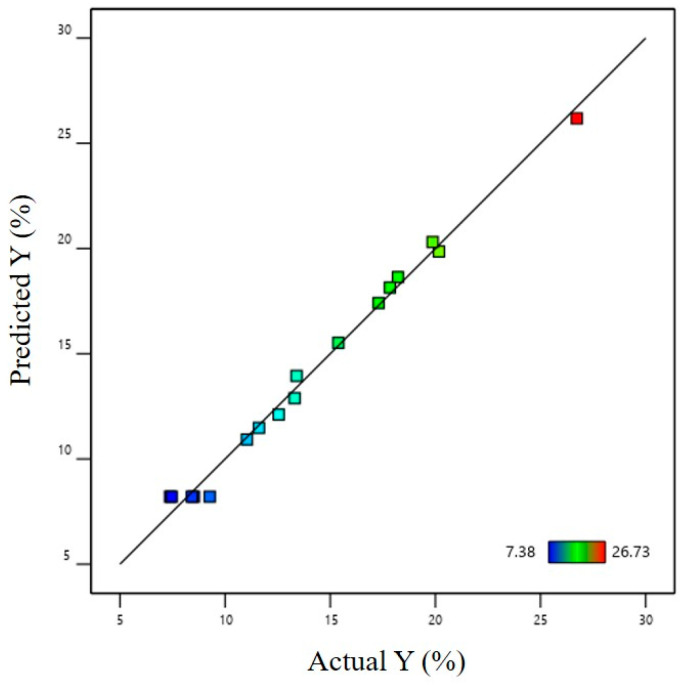
Distribution of actual and predicted values of maximum thinning rate.

Figure 6 shows the distribution result of the actual and predicted values of the maximum thinning rate of the hot-stamped sheets. The predicted and actual values are evenly distributed on the same line, indicating that this model has a certain forecasting ability.

### 3.3. Response Surface Analysis of Regression Models

**Figure 7:**
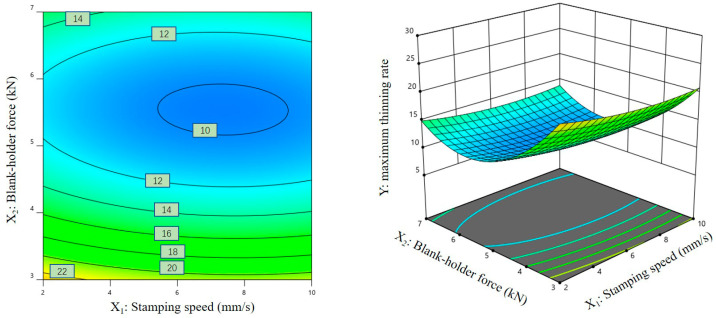
Contour plot and three-dimensional response surface graph of X_1_-X_2_ on Y.

**Figure 8:**
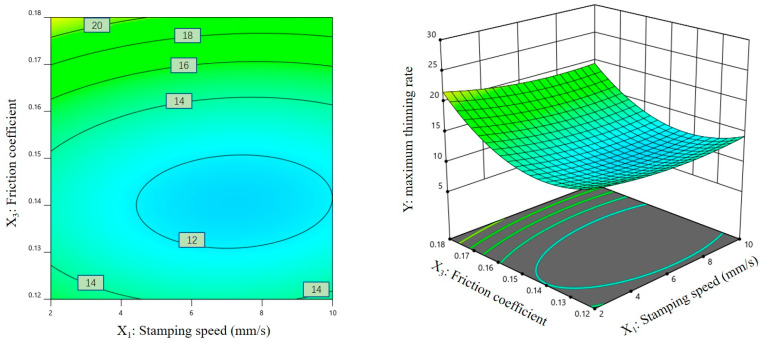
Contour plot and three-dimensional response surface graph of X_1_-X_3_ on Y.

**Figure 9:**
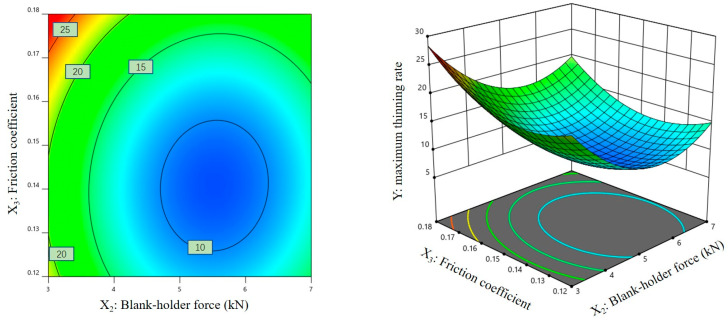
Contour plot and three-dimensional response surface graph of X_2_-X_3_ on Y.

Figure 7, Figure 8 and Figure 9 show the contour plots and corresponding three-dimensional response surface graphs of the interactions among response factors when the maximum thinning rate of the magnesium alloy sheet was taken as the response variable. Figure 7 and Figure 8 show that the contours on the Y plane are dense, and the contours corresponding to the changes in X_1_ and X_2_/X_3_ reveal elliptic characteristics. These results show that there was a significant interaction between the stamping speed and the blank-holder force, as well as between the stamping speed and the friction coefficient.

Figure 9 shows that the contour lines on the Y plane are sparse and appear as regular circles. The slope of the response surface is steeper when one of the response factors changes, and the other is gentler when the response factor changes, which indicates that the interaction between the blank-holder force and the friction coefficient was not significant in the simulation results of the maximum thinning rate of the hot-stamped sheet.

According to the response surface optimization analysis, the optimal configuration of process parameters was as follows: The stamping speed was 7.067 mm/s, the blank-holder force was 5.533 kN, and the friction coefficient was 0.142. Under these parameters, the optimal maximum thinning rate of the hot-stamped sheet was 7.37%.

## 4. Experimental Verification of Hot-Stamping Process

Further experiments on the hot stamping of magnesium alloy sheets were conducted to verify the forming parameters of the sheet reported in Table 2. The equipment used in the stamping forming test was a YT32-200C four-column hydraulic press, with a nominal force of 20 kN and a maximum slide stroke of 710 mm. Before the test, the magnesium alloy sheet was put into the external resistance furnace, heated up and kept for 10 min, and then quickly transferred to the die. Three groups of parameters were randomly selected (i.e., Run 4, Run 7, and Run 13). The hydraulic press stroke was set so that the punch was pressed down to the specified position for the stamping test. Then, the thickness of the sheet after hot stamping (region D in Figure 4) was measured using a Doppler ultrasonic detector.

**Figure 10:**
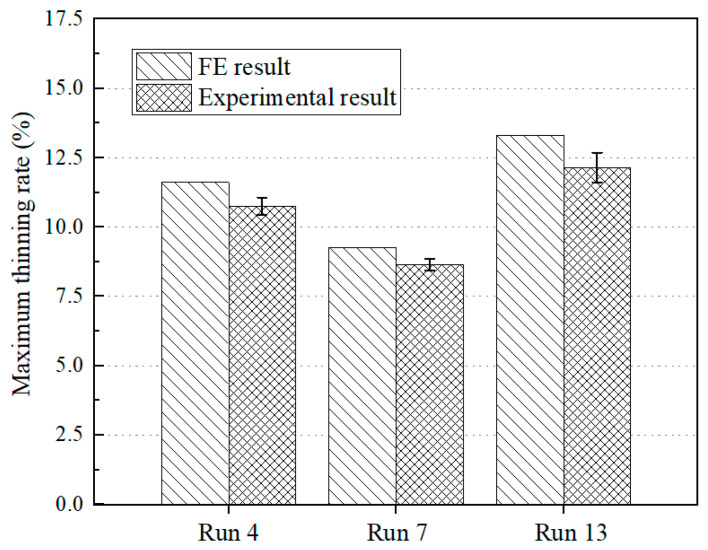
Comparison between simulation and testing results on the maximum thinning rate of the hot-stamped sheet.

A comparison of the maximum thinning rates obtained through simulation and testing under the same process parameters was carried out, and the comparative results are presented in Figure 10. The maximum relative error between the simulation and test values for the maximum thinning rate was 8.72%. This may be due to the absence of material damage parameters in the numerical model or the slip error between the fixture and the sheet in the test, which led to some differences between them. Additionally, this also showed that the combined method of the finite element model and response surface analysis had a high accuracy in the study of the magnesium alloy sheet’s formability.

This work attempts to provide a reliable methodology for the optimal design of the forming process in order to produce the required parts through hot stamping in a limited number of experiments. It was proved that the response surface method combined with the finite element method can be applied to optimize the process parameters of hot stamping. By improving the precision of the numerical model and optimizing the process parameters, the thinning of hot-stamped sheets can be minimized.

## 5. Conclusions

In this paper, a constitutive model suitable for describing magnesium alloy sheet forming was introduced. The hot-stamping process of an AZ31 magnesium alloy sheet was numerically analyzed based on the finite element solver ABAQUS/Explicit. Then, the response surface methodology was used to study the influence of the key process parameters on the maximum sheet thinning rate, and the optimal combination of the hot-stamping process parameters was obtained. Finally, the accuracy of the numerical model and the response surface model was verified by hot-stamping experiments. The main conclusions are summarized as follows:

(1) Numerical simulation results showed that the sheet near the corner region of the die was prone to thinning due to its large strain. With the established model, the numerical simulation of the hot-stamping process of the AZ31 alloy sheet could be achieved with a high level of accuracy.
(2) Through analyzing the variance results of the maximum thinning rate obtained using the response surface methodology, it can be concluded that each of the process parameters affected the maximum thinning rate in the order of blank-holder force (X_2_) > friction coefficient (X_3_) > stamping speed (X_1_). Hot-stamped sheets’ maximum thinning rate largely depended on the interaction between X_1_ and X_2_/X_3_.
(3) According to an optimization analysis using the response surface methodology, the optimal process parameters for the hot-stamped AZ31 magnesium alloy sheet were as follows: The stamping speed was 7.067 mm/s, the blank-holder force was 5.533 kN, and the friction coefficient was 0.142. Additionally, the maximum relative error (8.72%) was within a reasonable range after comparing the hot-stamping experiments and simulations.

This study can provide effective guidance for the process improvement and parameter optimization of the hot stamping of magnesium alloy sheets.

## Figures and Tables

**Table 1:** Chemical composition of AZ31 magnesium alloy (mass %).

Element	Mg	Al	Mn	Zn	Fe	Others
Value	94	3.1	0.31	0.58	0.004	0.36

**Table 2:** Hot-stamping process parameters of AZ31 magnesium alloy sheet.

Density (kg/m^3^)	Poisson Ratio	Thermal Expansion Coefficient (°C^−1^)	Thermal Conductivity (W/m °C)	Specific Heat (J/kg °C)
1780	0.33	2.75 × 10^−5^	98	1170

**Table 3:** Factors and levels for hot-stamping simulation.

Factor	Variable	Level		
		−1	0	+1
Stamping speed/*v* (mm/s)	X_1_	2	6	10
Blank-holder force/*F* (kN)	X_2_	3	5	7
Friction coefficient/*μ*	X_3_	0.12	0.15	0.18

**Table 4:** Experimental arrangement and response from hot-stamping simulation.

Run	Coded Level			Real Level			Y/%
	X_1_	X_2_	X_3_	*v* (mm/s)	*F* (kN)	*μ*	
1	0	−1	1	6	3	0.18	26.73
2	1	0	−1	10	5	0.12	11.04
3	−1	0	1	2	5	0.18	17.3
4	−1	0	−1	2	5	0.12	11.61
5	0	0	0	6	5	0.15	7.38
6	1	−1	0	10	3	0.15	18.22
7	0	0	0	6	5	0.15	9.27
8	1	0	1	10	5	0.18	15.39
9	0	−1	−1	6	3	0.12	20.17
10	0	1	1	6	7	0.18	17.84
11	0	0	0	6	5	0.15	8.52
12	1	1	0	10	7	0.15	12.55
13	−1	1	0	2	7	0.15	13.31
14	0	1	−1	6	7	0.12	13.4
15	−1	−1	0	2	3	0.15	19.87
16	0	0	0	6	5	0.15	7.46
17	0	0	0	6	5	0.15	8.42

**Table 5:** Variance analysis (ANOVA) for the maximum thinning rate of the stamping sheet.

Source	Statistical Analysis					
	Sum of Squares	df	Mean Square	*F*-Value	*p*-Value	Significant
Model	463.39	9	51.49	87.87	<0.0001	*
X_1_	2.99	1	2.99	5.1	0.0585	
X_2_	97.23	1	97.23	165.94	<0.0001	*
X_3_	55.34	1	55.34	94.44	<0.0001	*
X_1_ X_2_	0.198	1	0.198	0.338	0.5792	
X_1_ X_3_	0.4489	1	0.4489	0.7661	0.4104	
X_2_ X_3_	1.12	1	1.12	1.92	0.2087	
X_1_^2^	4.54	1	4.54	7.75	0.0271	
X_2_^2^	191.2	1	191.2	326.32	<0.0001	*
X_3_^2^	88.56	1	88.56	151.15	<0.0001	*
Residual	4.1	7	0.5859			
Lack of Fit	1.59	3	0.5288	0.841	0.5382	not sig.
Pure Error	2.52	4	0.6288			
Cor Total	467.49	16				

Note: R^2^ = 0.9912, R^2^(Adj) = 0.9799, R^2^(Pre) = 0.9373, Adeq precision (S/N) = 30.6107. “*” indicates model term is highly significant (*p* < 0.01).

## Data Availability

Not applicable.
